# A Case of Epididymo-orchitis after intravesical bacille Calmette-Guérin therapy for superficial bladder carcinoma in a patient with latent tuberculosis infection

**DOI:** 10.1186/s13027-016-0072-y

**Published:** 2016-06-02

**Authors:** C. Colomba, P. Di Carlo, G. Guadagnino, L. Siracusa, M. Trizzino, C. Gioè, A. Cascio

**Affiliations:** Dipartimento di scienze per la promozione della salute e materno-infantile, Università di Palermo, via del vespro 129, Palermo, 90127 Italy

**Keywords:** Calmette, Guérin, Intravesical, Bladder, Complications

## Abstract

**Background:**

Intravesical instillation of bacille Calmette-Guérin (BCG) has been established as efficient therapy for superficial bladder carcinoma. Overall, intravesical BCG is well tolerated and results in complications of less than 5 %. However, adverse effects such as granulomatous prostatitis, pneumonitis, hepatitis, sepsis, and hypersensitivity reactions may occur. The reported rate for tuberculous orchitis after BCG intravesical therapy is 0.4 %.

**Findings:**

We report a case of monolateral tuberculous orchitis occurring one month after the second course of intravescical instillation of bacille Calmette-Guérin in a patient with proven superficial bladder carcinoma and latent tuberculosis infection.

**Conclusions:**

In our opinion intravesical instillation of BCG should be considered on an individual patient basis, with full patient disclosure of the potentially significant risks. A screening with an intradermal Mantoux before starting the first cycle of BCG instillation should be recommended and isoniazid would be indicated as the treatment for latent tuberculosis infection.

## Introduction

Intravesical instillation of bacille Calmette-Guérin (BCG) has been established as efficient therapy for superficial bladder carcinoma. Intravesical BCG delays tumor progression and has been shown to eradicate residual tumors in 60 % of patients with papillary carcinoma and in 70 % of patients with carcinoma in situ [1–4]. Overall, intravesical BCG is well tolerated and results in complications of less than 5 %. However, adverse effects such as granulomatous prostatitis, pneumonitis, hepatitis, sepsis, and hypersensitivity reactions may occur [5]. Complications can appear early (within 3 months after instillation) or years after the first BCG treatment. Early-presentation disease is characterized by generalized symptoms, pneumonitis and hepatitis. Late-presentation disease is usually localized, with no systemic manifestations and the infection involves often the genitourinary tract and/or other sites that are typical for reactivation of mycobacterial disease, such as the vertebral spine or the retroperitoneal tissues. The reported rate for tuberculous orchitis after BCG intravesical therapy is 0.4 % [5]. We report a case of monolateral tuberculous orchitis occurring one month after the second course of intravesical instillation of BCG in a patient with proven superficial bladder carcinoma and latent tuberculosis infection.

## Case report

A 69-year-old patient came to Infectious Disease Unit of the “P. Giaccone” Teaching Hospital in Palermo, Italy, because of painful and progressive monolateral scrotal swelling. Two years earlier the patient underwent transurethral resection of a papillary carcinoma of the bladder, followed, after about two weeks from surgery, by a course of intravesical BCG therapy (six instillations weekly). The following year, during cystoscopy follow-up, a second course of BCG intravesical therapy was required due to a doubtful recurrence. After about six weeks from the end of the second therapy course, control cystoscopy and irrigation cytology were performed and showed no evidence of bladder carcinoma and normal urine cytology (Papanicolaou II). Nevertheless, maintenance dose of intravesical BCG therapy (one instillation every three weeks for about one-three years) was made and one month after the last instillation the patient began to complain of scrotal swelling. The patient had a medical history of type 2 diabetes and ulcerative rectocolitis on treatment with metformin and mesalazine respectively. On admission, the temperature was 36.9 °C, the blood pressure 130/70 mmHg, the pulse 100 beats per minute, the respiratory rate 18 breaths per minute and the oxygen saturation 96 % while he was breathing ambient air. The urine culture was negative. The left testis contained a soft painful mass with a cutaneous fistula; an ultrasound scan showed the presence of an abscess and surgery was required. Left orchiectomy and funiculus spermaticus ligature were performed, specimens were sent for histopathology and microbiological tests (bacterial culture). Histological investigation on testis showed a chronic granulomatous disease presenting with giant cells Langhans type, caseous necrosis and purulent abscess overlap. A computed tomography of the chest showed bilateral apical fibrosis and the presence of millimeter nodules in the right pulmonary lobe. An Interferon-Gamma Release Assay (QuantiFERON-TB Gold, Cellestis, Ltd., Carnegie, Australia) was performed and immune reactivity to *Mycobacterium tuberculosis* (Mtb) was proved whereas three sputum and urine sample examination with the Ziehl Neelsen (ZN) technique and Polymerase Chain Reaction (PCR) for *Mycobacterium tuberculosis* were negative. Because of the BCG instillations therapy history, treatment with rifampicin 600 mg daily, isoniazid 300 mg daily and ethambutol 1500 mg daily was started. Therefore patient was discharged with indication to continue antituberculosis therapy for six month of isoniazid plus rifampicin with a two month of intensive phase including ethambutol.

## Discussion

The intravesical administration of BCG has become a mainstay of adjunctive therapy for superficial bladder cancer. Although usually well tolerated, both local and systemic BCG-related complications may occur following instillation. These events are uncommon (<5 %) and the majority of patients tolerate BCG therapy without significant morbidity [1–6]. The mechanism of action of BCG in bladder cancer is still unclear and probably is specific anti-BCG cell-mediated immunity. BCG triggers a variety of local immune responses that appear to correlate with antitumor activity. Studies of the immunological mechanism of BCG therapy show that an intact immune system, particularly the cellular system, is required for antitumor activity because mycobacterial antigen presentation by phagocytes to T helper cells is the pivotal interaction [7]. The pathogenic mechanisms underlying the development of complications following BCG instillation remain not fully understood. Most of the symptoms associated with BCG immunotherapy are the result of the immune stimulation that is required to effectively eradicate cancer cells or are an ongoing active infection. These symptoms include urinary frequency and burning, mild malaise, and low-grade fever. Disseminated infection is the most common manifestation. This complication consist of miliary tuberculosis, fever associated to bone marrow and/or liver infiltration, sepsis with multiorgan failure, or persistent fever as the unique clinical manifestation responding to antituberculosis treatment [8–10]. Localized and late infection can involve osteoarticular apparatus (spondylodiscitis), muscles, vessels (aneurysms and pseudoaneurysms), eyes (granulomatous anterior uveitis, endophthalmitis, autoimmune retinopathy) and genitourinary tract (cystitis and sterile pyuria, diffuse prostatic enlargement or focal nodule or abscess, nephritis, epididymo-orchitis) [5, 10, 11]. Our patient developed epididymo-orchitis without generalized symptoms one month after the second course of six weekly intravesical instillation of BCG. It is known that diabetic patients have a greater risk of developing tuberculosis and that poor glycemic control is associated with complications from intravesical BCG [12]. However, our patient has a well-compensated diabetes. The patient did not report history of tuberculosis but a computed tomography scan showed nodular lesions in the right pulmonary lobe and he had a positive QuantiFERON-TB Gold test. Its positivity only indicates previous contact with *Mycobacterium tuberculosis* and it is not useful for the diagnosis of BCG complications. Several studies demonstrated that the antitumor effect of BCG is not immediate but is the consequence of prior immune stimulation and for this reason some patients do not respond to an initial 6-week course of BCG and need an additional 6-week course of BCG to become tumor-free [13]. A previous tuberculosis or a latent tuberculosis infection do not seem to be associated with a higher risk of BCG complications. Other authors found no significant differences in terms of previous diagnosis of active or latent tuberculosis infection between patients with and without systemic BCG infection. Mucosal disruption of the urogenital epithelium caused by difficult bladder catheterizations, preexisting cystitis or persistent hematuria following transurethral resection of the bladder tumor, is the main predisposing factor [10]. Concurrent prophylactic use of isoniazid with intravesical instillation has largely been unsuccessful in the protection against BCG complication. On the contrary prophylactic use of ofloxacin may be more effective [14, 15]. A screening with an intradermal Mantoux before starting the first cycle of BCG instillation should be recommended. Active tuberculosis has to be ruled out and isoniazid would be indicated as the treatment for latent tuberculosis infection. Patient monitoring is important for the diagnosis and early treatment of BCG therapy related complications.
